# Leisure-time, occupational, and commuting physical activity and the risk of chronic kidney disease in a working population

**DOI:** 10.1038/s41598-021-91525-4

**Published:** 2021-06-10

**Authors:** Shohei Yamamoto, Yosuke Inoue, Keisuke Kuwahara, Takako Miki, Tohru Nakagawa, Toru Honda, Shuichiro Yamamoto, Takeshi Hayashi, Tetsuya Mizoue

**Affiliations:** 1 Department of Epidemiology and Prevention, Center for Clinical Sciences, National Center for Global Health and Medicine, Toyama, 1-21-1, Shinjuku-ku, Tokyo, 162-8655 Japan; 2 Depertment of Rehabilitation Sciences, Kitasato University Graduate School of Medical Sciences, Kanagawa, Japan; 3 Teikyo University Graduate School of Public Health, Tokyo, Japan; 4 Hitachi Health Care Center, Hitachi, Ltd, Hitachi, Ibaraki Japan

**Keywords:** Chronic kidney disease, Epidemiology, Lifestyle modification

## Abstract

Physical activity has been linked to a lower risk of chronic kidney disease (CKD); however, evidence on the relationship between domain-specific physical activity and CKD is scarce. This study aimed to examine the risk of CKD in relation to leisure-time, occupational, and commuting physical activities in a large occupational cohort in Japan. Participants were 17,331 workers (20–65 years old) without CKD and were followed-up for a maximum period of 13 years. Incident CKD was defined as an estimated glomerular filtration rate of < 60 mL/min/1.73 m^2^ and/or proteinuria determined using the dipstick test. The Cox proportional hazards models were used to examine the associations. During 147,752 person-years of follow-up, 4013 participants developed CKD. Workers who were standing or walking at work and those who were fairly active at work had adjusted hazard ratios of 0.88 (95% confidence interval 0.86–0.96) and 0.89 (95% confidence interval 0.78–1.02), respectively, for developing CKD than sedentary workers. Leisure-time physical activity and walking for commute were not associated with CKD risk. Our findings suggest that occupational, but not leisure-time and commuting physical activities, is associated with a lower CKD risk.

## Introduction

Chronic kidney disease (CKD) is a major clinical and public health problem; it is a precursor for end-stage renal disease and a strong risk factor for cardiovascular morbidity and mortality^1,2^. In 2017, the global prevalence of CKD was 9.1%, which was equivalent to approximately 700 million cases^1^. Few effective treatments are available to treat patients with CKD; thus, the focus has been on the prevention of CKD incidence^3,4^. According to the literature, CKD shares several established risk factors (i.e., diabetes, hypertension, and obesity) with cardiovascular diseases^5^.

Physical activity is another important factor for reducing cardiovascular risk^6^ and is hypothesized to be an important modifiable risk factor in the development of CKD, either directly or indirectly, with favorable effects on diabetes, hypertension, and obesity^7,8^. A recent meta-analysis of nine cohort studies showed that higher physical activity levels were associated with a lower risk of CKD^9^.

Several issues remain to be addressed for the association between physical activity and CKD incidence. First, although workers spend much of their day at work and the majority of their daily physical activity^10^ and sedentary behavior^11^ occur in the workplace, few studies have focused on occupational physical activity^12,13^. Prolonged sitting time has been linked to impaired insulin sensitivity and lipid metabolism, and elevated inflammation^14,15^, all of which may contribute to the development of CKD^5^. Second, it remains unknown whether the physical activity-CKD association differs according to health status that has been proposed to increase the risk of CKD (i.e., diabetes, hypertension, or obesity). Such data are essential for revealing the biological interaction between physical activity and these health conditions concerning CKD onset. Finally, epidemiological data on these issues are limited among the Japanese population^12^, which has one of the highest prevalence of CKD^16,17^ and insufficient rates of physical activity^18^.

To address these issues, we prospectively investigated the associations of leisure-time, occupational, and commuting physical activities with the risk of CKD incidence in a large-scale cohort of the Japanese working population. We also analyzed the potential effect modification by a particular disease or condition or the other domains of physical activity based on the relationship between the three domains of physical activity and CKD.

## Methods

### Study setting

The study was conducted within the Japan Epidemiology Collaboration on Occupational Health (J-ECOH) study^19^, an ongoing large-scale cohort study of workers. Of the participating companies, one company manufacturing electrical machinery and apparatus provided check-up data that included detailed information on physical activity, which forms the basis of the present analysis.

Prior to data collection, the conduct of the study was announced using a poster, informing that health-related data of employees owned by the company (i.e., health check-ups, cardiovascular disease events, long-term sick leave, and death) would be anonymized and provided to the study. While participants did not provide written or verbal informed consent, they were allowed to refuse or withdraw their participation at any time. This opt-out procedure conforms to the Japanese Ethical Guidelines for Epidemiological Research for observational studies that use existing data. The study protocol, including the consent procedure (informed consent waiver), was approved by the ethics committee of the National Center for Global Health and Medicine, Japan (approval number: NCGM-G-001140). All methods were carried out in accordance with the ethical standards of the 1975 Declaration of Helsinki, as revised in 2013.

### Study cohort

Overall, 23,248 workers, aged 20–65 years, received health check-ups (comprehensive type) between April 2006 and March 2007 (baseline period) and had data for serum creatinine. We excluded workers with CKD (defined as an estimated glomerular filtration rate [eGFR] of < 60 mL/min/1.73 m^2^ and/or proteinuria [≥ 1+ on dipstick^20^]; n = 1510), with an eGFR of ≥ 200 mL/min/1.73 m^2^ (owing to possible measurement errors^21^; n = 5), incomplete information on physical activity (n = 2424), and engagement in an unspecified activity only during leisure (n = 489), and with self-reported cancer (n = 179). Workers who did not attend subsequent health check-ups or who had no measurement of eGFR or proteinuria in a subsequent health check-up (n = 1973) were excluded. Finally, 17,331 participants (15,544 men and 1787 women) were included in the analyses (Supplementary Fig. S1).

### Assessment of physical activity on leisure, work, and commuting

We assessed leisure-time, occupational, and commuting physical activity using a questionnaire that was designed specifically for health check-ups of the participating company. This questionnaire was not validated but has a structure similar to the previously validated and reproducible ones^22,23^.

Regarding leisure-time physical activity, participants were asked to choose up to three activities among a list of 20 exercise or sports activities and the frequency (times per month) and duration per occasion (minutes) for each activity. If participants engaged in activities not listed in the questionnaire, they were instructed to choose an activity of similar intensity from the list. Of the 20 exercise or sports activities in the list, the activity named ‘‘Other’’ was not used for further analysis.

The metabolic equivalent (MET; 1 MET = 1 kcal per h per kilogram of body weight) values for each activity were determined according to Ainsworth’s compendium of physical activities^24^. Of the 19 activities, 12 (walking not for work or commuting, walking fast not for work or commuting, golf practice, golf, baseball, softball, bike cycling, table tennis, pang-pong, badminton, muscle strength training, and radio gymnastics) were classified as moderate activities (3–5.9 MET), and seven (light jogging [approximately 6 min/km], jogging, swimming, soccer, tennis, aerobics, and jump rope) were classified as vigorous activities (≥ 6 MET). Leisure-time physical activity was defined as the product of intensity (MET) and duration of exercise (h), and the calculated MET-h/week of each individual was placed into one of four categories: inactive (0 MET-h/week), low (> 0 to < 7.5 MET-h/week), moderate (≥ 7.5 to < 16.5 MET-h/week), or high (≥ 16.5 MET-h/week). This classification corresponds to the cut-off points in the physical activity guidelines for Americans^25^. Although the guideline refers to any physical activity, not just leisure-time physical activity, this classification has been used in previous studies with leisure-time physical activity^26–28^.

Occupational physical activity was assessed using a single-item question “To what extent is your work physically demanding?” with the following response options: “mostly sedentary”, “mostly standing”, “walking often”, or “fairly active.” In the analyses, we combined “mostly standing” and “walking often” because standing and walking in an occupational setting are moderate-intensity activities (3–5 METs)^24^.

Commuting physical activity was assessed using the self-reported duration of walking to and from work (in minutes) and was categorized as < 20, 20 to < 40, and ≥ 40 min for the analysis, similar to other studies in Japan^27,29^.

### Ascertainment of chronic kidney disease cases

CKD was assessed using the data of annual health check-ups from baseline to March 2019 and was defined as an eGFR of < 60 mL/min/1.73 m^2^ and/or with proteinuria (≥ 1+ on dipstick), according to the clinical practice guideline for CKD (KDIGO)^5^. eGFR was calculated using the following formula established by the working group of the Japanese CKD Initiative^20^: eGFR (mL/min/1.73 m^2^) = 1.94 × (serum creatinine level)^−1.094^ × (age)^−0.287^ × (0.739 if women)^30^. Serum creatinine level was measured using the enzyme method with an autoanalyzer (Hitachi 7600, Japan). Proteinuria was tested using dipsticks and an autoanalyzer (Siemens Healthcare, Japan) and was categorized as negative, ±, 1+, 2+, and 3+ (corresponding to protein levels of undetectable, trace, 30 mg/dL, 100 mg/dL, and ≥ 300 mg/dL, respectively). The date of check-up when CKD was first identified was the incidence date of CKD.

### Covariates

The covariates included eGFR, age, sex, smoking status, alcohol consumption, occupation, job position, overtime work, shift work, commuting mode, sleep duration, hypertension, diabetes, history of cardiovascular disease, dyslipidemia, hyperuricemia, and body mass index (BMI) at baseline. We refer to the online Supplementary Appendix 1 for data collection methods, which have been described in the previous studies^27,31,32^.

### Treatment of missing data

The proportion of missing data for each covariate was as follows: smoking status (0.4%), occupation (3.1%), job position (3.3%), monthly overtime work (3.6%), shiftwork (2.9%), primary commuting mode (0.1%), sleep duration (0.2%), diabetes (0.2%), dyslipidemia (0.02%), and hyperuricemia (0.04%).

Multiple imputations were performed using the chained equation method, with multinomial logistic regression performed for imputation of smoking status, occupation, monthly overtime work, primary commuting mode, and sleep duration and logistic regression performed for the job position, shiftwork, diabetes, dyslipidemia, and hyperuricemia. The imputation used all the variables involved in all the analytic models, including the outcome variables of time-to-event and event status. The 20 imputed datasets were generated, and the results were combined using Rubin’s rules.

### Statistical analyses

We calculated person-years of follow-up for each participant from the date of the baseline health check-up to the date of health check-up when CKD was first identified or the date of the last health check-up, whichever came first. We conducted the Cox proportional hazards regression to calculate hazard ratios (HRs) and 95% confidence intervals (CIs) for the association between leisure-time, occupational, and commuting physical activities and the risk of CKD.

Covariates were adjusted in a stepwise manner. In model 1, we adjusted for age (continuous, years), sex, and baseline eGFR. In model 2, we additionally adjusted for smoking status (never, former, or current), alcohol consumption (0, > 0 to < 2, or ≥ 2 go/day), occupation (engineering, production, office and administration, or others), job position (high or low), overtime work (< 45, 45 to < 60, 60 to < 80, 80 to < 100, or ≥ 100 h/month), shift work (yes or no), primary commuting mode (walking, bicycling, train/bus, or car/motorbike), sleep duration (< 5, 5 to < 6, 6 to < 7, or ≥ 7 h per day), and other domains of physical activity. In model 3, we further adjusted for potential mediators, such as hypertension, diabetes, history of cardiovascular disease, dyslipidemia, hyperuricemia, and BMI (< 18.5, 18.5 to < 25.0, 25.0 to < 30.0, or ≥ 30.0 kg/m^2^).

The trend association between leisure-time physical activity and risk of CKD was assessed by assigning the median dose of leisure-time physical activity in each category and treating this variable as continuous. For the trend for occupational physical activity, a score of 1–3 was assigned to sedentary work, standing or walking during work, and fairly active during work, respectively. For commuting physical activity, we assigned 10, 30, and 50 min to increasing categories of walking to and from work (< 20, 20 to < 40, and ≥ 40 min).

To test the effect modification by sex, age (< 50 or ≥ 50 years), hypertension, diabetes, obesity (BMI < 25 or ≥ 25 kg/m^2^), baseline eGFR (60–89 or ≥ 90 mL/min/1.73 m^2^), and occupational (sedentary work or not) and commuting physical activities (< 20 or ≥ 20 min), we included the interaction term between each variable and leisure-time physical activity one-by-one in the fully adjusted model (Model 3) and conducted Wald tests for interactions. These subgroup analyses were repeated between occupational or commuting physical activity and CKD onset. The proportional hazard assumption was examined using the Schoenfeld residuals, and all covariates agreed with the proportional hypothesis, except for age and baseline eGFR.

We also conducted sensitivity analyses and excluded participants with < 2 years of follow-up for the aforementioned major analyses. To test the robustness of the association with leisure-time physical activity, the analyses were repeated using the cut-off points corresponding to the World Health Organization (WHO) for total physical activity^33^: inactive (0 MET-h/week), low (> 0 to < 7.5 MET-h/week), moderate (≥ 7.5 to < 15.0 MET-h/week), or high (≥ 15.0 MET-h/week).

All statistical analyses were performed using Stata/MP version 16.1 (Stata Corp., College Station, Texas, USA). Statistical significance was set with a two-sided P-value of 0.05 for all analyses.

## Results

At baseline, the mean age of 17,331 participants was 42.8 ± 10.0 years, and 90% were men; 64% of the participants were physically inactive during leisure, and 54% were sedentary during work. The characteristics of the study population according to baseline leisure-time physical activity categories are presented in Table 1. The proportions of men and those with diabetes were higher among participants with higher leisure-time physical activity. In contrast, the level of baseline eGFR and the proportions of current smokers and those with long overtime work decreased with increasing leisure-time physical activity. The baseline characteristics stratified by occupational or commuting physical activity are presented in Supplementary Tables S1 and S2.

**Table 1:** Baseline characteristics of the participants, according to the volume of leisure-time physical activity.

	Leisure-time physical activity (MET-h/week)			
	Inactive (0 MET-h/week)	Low (> 0 to < 7.5 MET-h/week)	Moderate (7.5 to < 16.5 MET-h/week)	High (≥ 16.5 MET-h/week)
Participants	n = 11,170	n = 2896	n = 1901	n = 1364
Male sex, n (%)	9826 (88.0)	2669 (92.2)	1763 (92.7)	1286 (94.3)
Age, years	42.7 ± 9.8	42.2 ± 10.0	43.1 ± 10.3	43.6 ± 10.9
eGFR, mL/min/1.73 m^2^	89.1 ± 16.1	87.3 ± 15.1	85.9 ± 15.1	85.8 ± 14.9
BMI, kg/m^2^	23.4 ± 3.5	23.6 ± 3.4	23.8 ± 3.2	23.6 ± 3.1
Smoking status, n (%)				
Never	3793 (34.0)	998 (34.5)	648 (34.1)	509 (37.3)
Former	1918 (17.2)	593 (20.5)	458 (24.1)	316 (23.2)
Current	5407 (48.4)	1298 (44.8)	791 (41.6)	537 (39.4)
Alcohol intake, n (%)				
None	3682 (33.0)	774 (26.7)	504 (26.5)	353 (25.9)
> 0 to < 2 go/day	5099 (45.6)	1513 (52.2)	955 (50.2)	673 (49.3)
≥ 2 go/day	2389 (21.4)	609 (21.0)	442 (23.3)	338 (24.8)
Occupation				
Engineering	3884 (34.8)	1011 (34.9)	666 (35.0)	444 (32.6)
Production	3473 (31.1)	918 (31.7)	550 (28.9)	421 (30.9)
Office and administration	1754 (15.7)	542 (18.7)	408 (21.5)	293 (21.5)
Other	1645 (14.7)	371 (12.8)	244 (12.8)	178 (13.0)
High job position, n (%)	1788 (16.0)	536 (18.5)	418 (22.0)	257 (18.8)
Long overtime work (≥ 45 h/month)	4000 (35.8)	1033 (35.7)	647 (34.0)	410 (30.1)
Shift work, n (%)	2929 (26.2)	723 (25.0)	426 (22.4)	328 (24.0)
Commuting mode, n (%)				
Walking	1578 (14.1)	392 (13.5)	257 (13.5)	191 (14.0)
Cycling	655 (5.9)	175 (6.0)	126 (6.6)	94 (6.9)
Bus/train (public transportation)	2439 (21.8)	596 (20.6)	359 (18.9)	234 (17.2)
Car/motorbike	6490 (58.1)	1731 (59.8)	1159 (61.0)	844 (61.9)
Sleep duration of < 6 h/day, n (%)	5708 (51.1)	1361 (47.0)	910 (47.9)	620 (45.5)
Hypertension, n (%)	1489 (13.3)	364 (12.6)	255 (13.4)	186 (13.6)
Diabetes, n (%)	802 (7.2)	242 (8.4)	173 (9.1)	123 (9.0)
History of CVD, n (%)	65 (0.6)	16 (0.6)	16 (0.8)	9 (0.7)
Dyslipidemia, n (%)	5111 (45.8)	1274 (44.0)	837 (44.0)	522 (38.3)
Hyperuricemia, n (%)	2218 (19.9)	596 (20.6)	388 (20.4)	262 (19.2)
Occupational physical activity, n (%)				
Sedentary	5867 (52.5)	1604 (55.4)	1130 (59.4)	726 (53.2)
Standing or walking	4095 (36.7)	989 (34.2)	594 (31.2)	472 (34.6)
Fairly active	1208 (10.8)	303 (10.5)	177 (9.3)	166 (12.2)
Commuting physical activity, n (%)				
0 to < 20 min	5979 (53.5)	1547 (53.4)	1070 (56.3)	786 (57.6)
20 to < 40 min	3557 (31.8)	912 (31.5)	543 (28.6)	405 (29.7)
≥ 40 min	1634 (14.6)	437 (15.1)	288 (15.1)	173 (12.7)

Data are shown as the mean ± standard deviation for continuous variables and as a number (percentages) for categorical variables. The proportion of missing data for each covariate was as follows: smoking status (0.4%), occupation (3.1%), job position (3.3%), monthly overtime work (3.6%), shiftwork (2.9%), primary commuting mode (0.1%), sleep duration (0.2%), diabetes (0.2%), dyslipidemia (0.02%), and hyperuricemia (0.04%).

*BMI* body mass index, *CVD* cardiovascular disease, *eGFR* estimated glomerular filtration rate.

During 147,752 person-years of follow-up, 4013 (23%) participants developed CKD, with an overall incidence rate of 27.2 cases per 1000 person-years. The median follow-up period was 10.6 years (range 0.2–13.0). Leisure-time physical activity was not significantly associated with the risk of developing CKD in any model (Table 2). An analysis using alternative cut-off points corresponding to the WHO also showed no association (Supplementary Table S3). In contrast, occupational physical activity was significantly associated with CKD risk in all models (Table 2). Relative to the participants who were sedentary during work and those who were standing or walking and fairly active during work had HRs of 0.88 [95% CI 0.81–0.96] and 0.89 [95% CI 0.78–1.02], respectively, in Model 2 (p for trend = 0.020). The association was virtually unchanged after additional adjustment for potential mediators (Model 3). Commuting physical activity was significantly associated with CKD in Model 1 (adjusted for age, sex, and baseline eGFR); however, the association was not statistically significant after adjusting for other covariates (Models 2 and 3). Subgroup analyses of leisure-time physical activity (Table 3) showed that the interactions by sex, age, hypertension, diabetes, obesity, and baseline kidney function were not statistically significant (p for interaction > 0.05). The HRs associated with leisure-time physical activity did not differ across the subgroups of other domains of physical activity, defined by occupational physical activity (sedentary or active during work) or commuting (< 20 min or ≥ 20 min for walking to and from work). Regarding the subgroup analyses of occupational and commuting physical activities, the interactions with other physical activity forms were null results (Supplementary Tables S4, S5).

**Table 2:** Hazard ratios of chronic kidney disease, according to leisure-time, occupational, and commuting physical activity.

	Cases/Subjects	Person-years	Model 1^b^	Model 2^c^	Model 3^d^
**Leisure-time physical activity**					
Inactive (0 MET-h/week)	2557/11,170	94,647	1.00 [reference]	1.00 [reference]	1.00 [reference]
Low (> 0 to < 7.5 MET-h/week)	643/2896	25,572	0.92 [0.84–1.00]	0.93 [0.85–1.01]	0.94 [0.86–1.03]
Moderate (7.5 to < 16.5 MET-h/week)	474/1901	16,116	1.04 [0.94–1.15]	1.05 [0.95–1.17]	1.06 [0.96–1.17]
High (≥ 16.5 MET-h/week)	339/1364	11,417	1.04 [0.92–1.16]	1.05 [0.94–1.18]	1.07 [0.96–1.20]
		*P* for trend^a^	0.402	0.255	0.112
**Occupational physical activity**					
Sedentary	2403/9327	79,584	**1.00 [reference]**	**1.00 [reference]**	**1.00 [reference]**
Standing/Walking	1246/6150	51,974	**0.88 [0.82–0.94]**^e^	**0.88 [0.81–0.96]**^e^	**0.88 [0.86–0.96]**^e^
Fairly active	364/1854	16,194	**0.88 [0.79–0.98]**^e^	**0.89 [0.78–1.02]**	**0.91 [0.81–1.03]**
		*P* for trend^a^	**< 0.001**	**0.020**	**0.015**
**Commuting physical activity**					
< 20 min	2013/9382	80,581	**1.00 [reference]**	1.00 [reference]	1.00 [reference]
20 to < 40 min	1334/5417	46,106	**1.10 [1.03–1.18]**^e^	1.06 [0.98–1.15]	1.06 [0.98–1.15]
≥ 40 min	666/2532	21,066	**1.14 [1.04–1.25]**^e^	1.08 [0.96–1.20]	1.08 [0.97–1.21]
		*P* for trend^a^	**0.001**	0.139	0.109

Data are shown as the hazard ratio [95% confidence interval].

^a^*P* value for the linear trend was calculated by using the Cox proportional hazards regression and assigning each category of physical activity as a continuous variable.

^b^Model 1 was adjusted for baseline estimated glomerular filtration rate (60 to 89 or ≥ 90 mL/min/1.73 m^2^), age (continuous), and sex.

^c^Model 2 was further adjusted for baseline smoking status (never, former, or current), alcohol consumption (0, > 0 to < 2, or ≥ 2go/day), occupation (engineering, production, office and administration, or others), job position (high or low), overtime work (< 45, 45 to < 60, 60 to < 80, 80 to < 100, or ≥ 100 h), shift work (yes or no), commuting mode (walking, bicycling, train/bus, or car/motorbike), sleep duration (< 5, 5 to < 6, 6 to < 7, or ≥ 7 h per day), and the other types of physical activity (i.e., leisure-time physical activity [0, > 0 to < 7.5, 7.5 to < 16.5, or ≥ 16.5 MET-h/week], occupational physical activity [sedentary, standing or walking, and fairly physically activity], or walking for commuting to and from work [< 20 min, 20 to < 40 min, or ≥ 40 min]).

^d^Model 3 was further adjusted for potential mediators, including baseline hypertension, diabetes, history of cardiovascular disease, dyslipidemia, hyperuricemia, and body mass index (< 18.5, 18.5 to < 25.0, 25.0 to < 30.0, or ≥ 30.0 kg/m^2^). ^e^*P* < 0.05 (2-tailed).

**Table 3:** Hazard ratios of chronic kidney disease according to the volume of leisure-time physical activity in subgroups.

Subgroups	Cases/Subjects	Person-years	Leisure-time physical activity (MET-h/week)				*P* for interaction
			Inactive (0 MET-h/week)	Low (> 0 to < 7.5 MET-h/week)	Moderate (7.5 to < 16.5 MET-h/week)	High (≥ 16.5 MET-h/week)	
**Sex**							
Men	3707/15,544	134,509	1.00 [reference]	0.95 [0.87–1.04]	1.06 [0.96–1.18]	1.06 [0.94–1.20]	0.815
Women	306/1787	13,243	1.00 [reference]	0.84 [0.58–1.22]	1.09 [0.71–1.66]	1.31 [0.81–2.11]	
**Age**							
< 50 years	2627/12,353	114,374	1.00 [reference]	0.95 [0.86–1.06]	1.03 [0.91–1.16]	1.10 [0.95–1.28]	0.913
≥ 50 years	1386/4978	33,378	1.00 [reference]	0.88 [0.75–1.03]	1.10 [0.93–1.29]	0.98 [0.81–1.17]	
**Hypertension**							
Yes	799/2294	15,989	1.00 [reference]	0.96 [0.79–1.18]	0.99 [0.80–1.24]	0.80 [0.61–1.06]	0.159
No	3214/15,037	131,764	1.00 [reference]	0.93 [0.85–1.03]	1.07 [0.96–1.19]	1.14 [1.00–1.28]	
**Diabetes**							
Yes	458/1340	9,338	1.00 [reference]	0.97 [0.76–1.25]	1.29 [1.00–1.75]	0.80 [0.55–1.16]	0.214
No	3543/15,956	138,141	1.00 [reference]	0.94 [0.85–1.03]	1.02 [0.92–1.14]	1.12 [0.99–1.27]	
**Obesity**							
BMI ≥ 25 kg/m^2^	1541/5054	41,180	1.00 [reference]	0.89 [0.77–1.02]	1.05 [0.90–1.23]	0.97 [0.80–1.18]	0.142
BMI < 25 kg/m^2^	2472/12,277	106,572	1.00 [reference]	0.97 [0.87–1.08]	1.06 [0.93–1.20]	1.14 [0.99–1.32]	
**Baseline eGFR**							
60 to 89 mL/min/1.73 m^2^	2843/10,248	84,227	1.00 [reference]	0.92 [0.83–1.02]	1.07 [0.95–1.20]	1.11 [0.97–1.26]	0.202
≥ 90 mL/min/1.73 m^2^	1170/7083	63,525	1.00 [reference]	0.98 [0.83–1.14]	1.04 [0.85–1.27]	0.93 [0.73–1.18]	
**Occupational physical activity**							
Sedentary	2403/9327	79,584	1.00 [reference]	1.01 [0.90–1.12]	1.14 [1.00–1.29]	1.07 [0.92–1.24]	0.596
Active^a^	1610/8004	68,168	1.00 [reference]	0.84 [0.73–0.97]	0.91 [0.77–1.08]	1.08 [0.90–1.28]	
**Commuting physical activity**							
< 20 min	2013/9382	80,581	1.00 [reference]	0.92 [0.81–1.05]	1.09 [0.95–1.25]	1.11 [0.95–1.29]	0.964
≥ 20 min	2000/7949	67,171	1.00 [reference]	0.96 [0.85–1.08]	1.03 [0.89–1.19]	1.04 [0.88–1.24]	

Data are shown as the hazard ratio [95% confidence interval].

*BMI* body mass index.

^a^Active including standing, walking, and fairly active at work.

The Cox proportional hazards regression adjusted for baseline age (continuous), sex, smoking status (never, former, or current), alcohol consumption (0, > 0 to < 2, or ≥ 2go/day), occupation (engineering, production, office and administration, or others), job position (high or low), overtime work (< 45, 45 to < 60, 60 to < 80, 80 to < 100, or ≥ 100 h), shift work (yes or no), primary commuting mode (walking, bicycling, train/bus, or car/motorbike), sleep duration (< 5, 5 to < 6, 6 to < 7, or ≥ 7 h per day), hypertension, diabetes, history of cardiovascular disease, dyslipidemia, hyperuricemia, body mass index (< 18.5, 18.5 to < 25.0, 25.0 to < 30.0, or ≥ 30.0 kg/m^2^), baseline estimated glomerular filtration rate (60–89 or ≥ 90 mL/min/1.73 m^2^), occupational physical activity (sedentary, standing or walking, and fairly physically activity), and walking for commuting to and from work (< 20 min, 20 to < 40 min, or ≥ 40 min).

In the sensitivity analyses excluding participants with < 2 years of follow-up (1550 participants, including 824 incident cases), the associations virtually did not change. That is, the HRs of standing or walking (0.87, 95% CI 0.79–0.95) and fairly active (0.87, 95% CI 0.76–1.00) were lower than those of sedentary activities during work (Supplementary Table S6).

## Discussion

In this large Japanese working population, we examined the associations between domain-specific physical activities and CKD onset. Physical activities on leisure and commuting were not associated with CKD risk, while standing or walking during work was associated with a lower CKD risk than sitting.

Previous studies have reported conflicting results on the association between leisure-time physical activity and CKD incidence. Some studies showed a lower CKD risk associated with higher leisure-time physical activity^28,34–38^; however, others did not^12,39–42^. One reason for this inconsistency may be ascribed to the difference in questionnaires used. Some studies^12,34–36,40^ assessed physical activity using only one or two items (for example, “Do you engage in habitual moderate physical activity for at least 30 min at a time, at least twice a week?”^36^), while others^28,39,42^ assessed the intensity, frequency, and duration of the physical activity. Nonetheless, results are conflicting even among studies using comprehensive questionnaires^28,39,42^. The inconsistency may be owing to the different backgrounds of the participants (i.e., patients with type 2 diabetes^35^, older participants^42^, men^36,40^, and workers^12,40^). Similar to our study, two studies on a working population reported no association^12,40^.

Epidemiological evidence linking CKD to occupational physical activity is scarce. We found a lower risk of CKD among participants who were mostly standing or walking during work than those who were mostly sitting during work. This finding accords with an observation among university staff in Japan, indicating a higher risk of proteinuria among sedentary male workers than non-sedentary counterparts^12^. These data suggest that moderate physical activity at work is beneficial for the maintenance of kidney function. We observed no further reduction in CKD risk associated with fairly active work, suggesting that vigorous activity at work does not provide additional benefits for CKD prevention. More studies with a detailed assessment of occupational physical activity are necessary to confirm our findings.

We observed no association between commuting physical activity and CKD incidence in participants and those engaging in sedentary work (Supplementary Table S5). This finding contrasts with the finding of a cross-sectional study in Northern Ireland, which showed that better renal function was associated with higher physical activity on commuting^13^. They assessed commuting physical activity using the information on intensity, frequency, ad duration of walking and cycling^13^, while our study was based exclusively on time spent in walking. In our subsidiary analysis for commuting mode, neither cycling (HR 1.03; 95% CI 0.89–1.19) nor walking (HR 1.06; 95% CI 0.95–1.18) was associated with a decreased risk of CKD than using car/motorbike (Supplementary Table S7).

One line of research on the association between physical activity and health outcomes has suggested differential effects of leisure-time (an inverse association) and occupational physical activity (a positive association) in relation to cardiovascular health and mortality^43^. This phenomenon termed “physical activity paradox^44^”, however, was not observed in our study. Similarly, the aforementioned study on a working population^12^ found that occupational physical activity, but not leisure-time physical activity, was associated with a lower risk of proteinuria. Additionally, the differential association of physical activity domains was not observed in relation to other health outcomes (e.g., diabetes^45^ and colon and breast cancers^46^). The “physical activity paradox” may thus depend on the outcome under study.

Our study has several strengths, for example, the large size of the cohort, long-term follow-up, and annual assessments with data on serum creatinine and proteinuria. Moreover, we adjusted for comprehensive covariates, including sleep duration and work-related factors, which the previous literature had not considered.

However, there are several limitations to this study. First, physical activity was assessed using an unvalidated questionnaire designed specifically for the company’s health check-up. We should note that the questionnaire used in this study is comparable to the validated ones. Specifically, our questionnaire on leisure-time physical activity is similar in structure to the questionnaire validated by Matthews et al.^22^, where respondents are asked to report up to three types of habitual exercise/sports and the duration and frequency per occasion of these activities; the difference from ours is that it specifies time frame [during the past 5 years]). Regarding occupational physical activity, our questionnaire resembles a previously validated one by Kurtzen et al.^23^, which is composed of single question with four response options (sedentary work, walk a lot, walk or lift a lot, heavy physical work). Second, we used only baseline information on physical activity, and the non-differential misclassification in physical activity owing to within-individual variability during follow-ups would lead to null results. Third, residual confounding and unmeasured confounders, such as diet, might have affected the risk of CKD. Fourth, we did not obtain data on socioeconomic status (e.g., income and education) other than job rank and occupation. Given that low socioeconomic status has been linked to both higher physical activity during work^47^ and a higher risk of CKD^48^, the incomplete adjustment of socioeconomic factors might have biased the association with occupational physical activity. Fifth, we defined an incident of CKD using a single point measurement of eGFR and/or proteinuria. Thus, because the clinical diagnosis of CKD is determined based on two parameters measured at least 3 months apart^20^, our criteria for CKD had a sensitivity of 100% but had low specificity, distorting the associations toward the null. Finally, our participants were mostly men (90%), and the study was restricted to workers in a company of the electrical machinery and apparatus manufacturing industry. Therefore, caution should be exercised when generalizing our findings to women or workers in other industries. We were unable to infer CKD risk for workers with extremely high occupational physical activity, such as farmers and construction laborers.

In conclusion, we demonstrated that occupational, but not leisure-time and commuting physical activities, was associated with a lower risk of CKD incidence in this Japanese large-scale working population.

## Supplementary Information

Supplementary Information.

## Data Availability

The datasets generated and/or analyzed during the current study are not publicly available due to ethical restrictions and participant confidentiality concerns, but de-identified data are available from Dr. Mizoue (Department of Epidemiology and Prevention, Center for Clinical Sciences, National Center for Global Health and Medicine, Tokyo, Japan) to qualified researchers on reasonable request.
