# Supplementary material for: Leisure-time, occupational, and commuting physical activity and the risk of chronic kidney disease in a working population
Source: Sci Rep. 2021 Jun 10;11:12308. doi: 10.1038/s41598-021-91525-4 (PMC8192894; doi:10.1038/s41598-021-91525-4)
Supplement: Supplementary file 1 — Supplementary Information. [file 41598_2021_91525_MOESM1_ESM.docx]

**Leisure-time, occupational, and commuting physical activity and the risk of chronic kidney disease in a working population**

Shohei Yamamoto, MSc^1,2, *^; Yosuke Inoue, PhD^1^; Keisuke Kuwahara, PhD^1,3^; Takako Miki, PhD^1^; Tohru Nakagawa, PhD^4^; Toru Honda, MD^4^; Shuichiro Yamamoto, PhD^4^; Takeshi Hayashi, PhD^4^; Tetsuya Mizoue, PhD^1^

**Affiliations:**

^1^Department of Epidemiology and Prevention, Center for Clinical Sciences, National Center for Global Health and Medicine, Tokyo, Japan.

^2^Depertment of Rehabilitation Sciences, Kitasato University Graduate School of Medical Sciences, Kanagawa, Japan.

^3^Teikyo University Graduate School of Public Health, Tokyo, Japan.

^4^Hitachi Health Care Center, Hitachi, Ltd, Ibaraki, Japan.

***Corresponding author:** Shohei Yamamoto


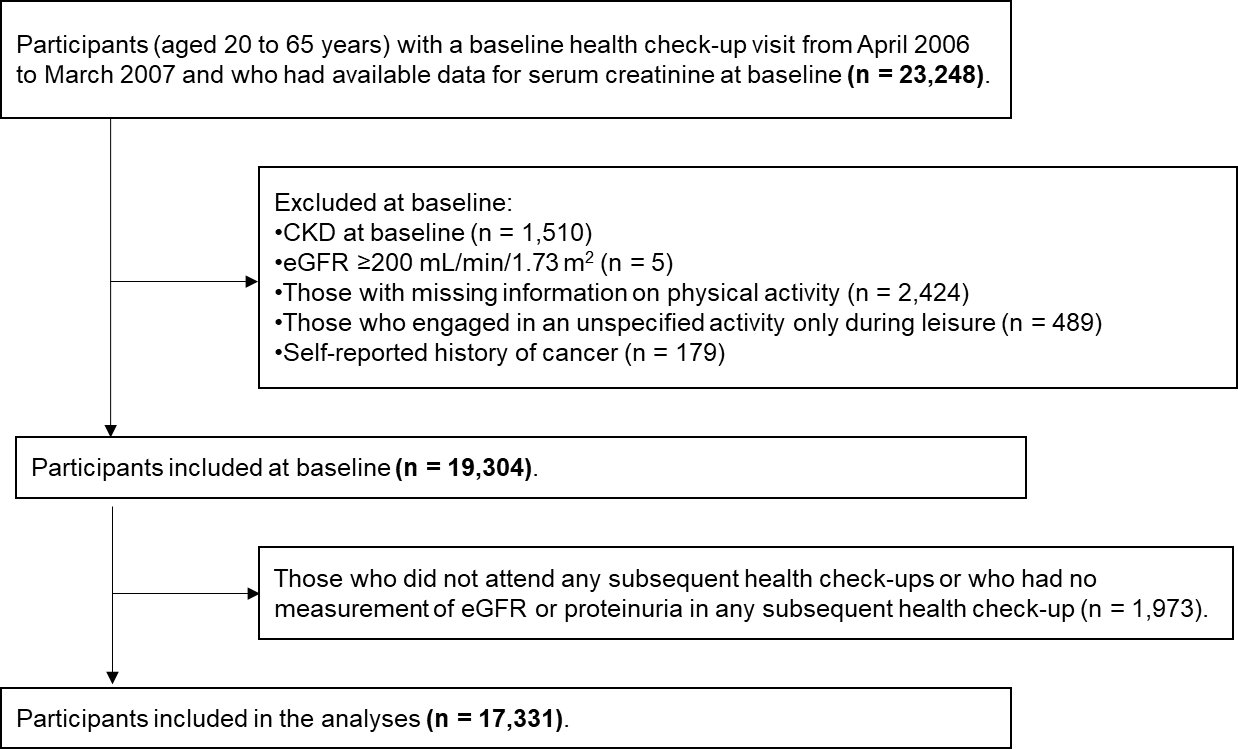


**Figure S1.** Flowchart of participant selection.

**Supplementary Appendix 1.** Data collection methods for covariates

**Covariates**

Body height and body weight was measured to the nearest 0.1 cm and 0.1 kg, respectively. Body mass index (BMI) was calculated as weight (kg) divided by height squared (m^2^). Blood pressures were assessed with an automated sphygmomanometer (BP-203RV III; Colin, Tokyo, Japan) in a sitting position after a 5-min rest. The high-density lipoprotein cholesterol (HDL-C) and low-density lipoprotein cholesterol (LDL-C) levels were measured by the direct method. The triglyceride levels were measured by the enzyme method. The plasma glucose was measured by the glucose oxidase peroxidative electrode method. HbA1c was measured by high-performance liquid chromatography.

Hypertension was defined as systolic blood pressure ≥140 mmHg, diastolic blood pressure ≥90 mmHg, or use of antihypertensive medications. Diabetes was defined as HbA1c≥6.5 % (48 mmol/mol), fasting blood glucose≥126 mg/dl (7.0 mmol/l), or receiving medical treatment for diabetes. Cardiovascular disease was defined as a self-reported history of myocardial infarction, angina, or stroke. Dyslipidemia was defined as triglyceride level ≥150 mg/dl, low-density lipoprotein cholesterol level ≥140 mg/dl, high-density lipoprotein cholesterol level <40 mg/dl, or receiving medical treatment for dyslipidemia. Hyperuricemia was defined as uric acid>7 mg/dl or receiving medical treatment for hyperuricemia.

Data on medical history, work-related factors, including occupation, job position, overtime work, and shift work, primary commuting mode, smoking, alcohol use, and sleep duration were ascertained by a standardized, self-administered questionnaire.

Participants were asked to choose their occupation from a list of 12 job categories, which we classified into engineering (design and development, inspection, and research), production (manufacturing and assembly), office and administrative (production and technology management, office and administrative, marketing, and data entry and information processing), and others (transportation and material moving; healthcare; customer service; and others).

The job position was categorized as high (department chief, department director, or higher position) or low (others). Monthly overtime work in the past 2–3 months was assessed with 1 question with five responses (<45, 45 to <60, 60 to <80, 80 to <100, or ≥100 h). Shift work was self-reported by using 1 question with three response options (shift work, nightshift work only, or no shift work). The primary commuting mode to work was self-reported according to 4 response options (walking, bicycling, train or bus, and car or motorbike). The total amount of alcohol consumption was estimated by using data on the frequency (number of days per week) and the amount of alcohol consumption of common beverages per day, as indicated by an equivalent amount of 1 unit (*go*) of Japanese sake. One *go* of Japanese sake contains approximately 23 g of ethanol. Sleep duration was measured with 1 question with four response options (<5, 5 to<6, 6 to<7, or ≥7 h per day).

**Table S1.** Baseline characteristics of participants according to occupational physical activity

|  | Occupational physical activity | | |
| --- | --- | --- | --- |
|  | Sedentary | Standing or Walking | Fairly Active |
| Participants | n = 9,327 | n = 6,150 | n = 1,854 |
| Male sex, n (%) | 8,556 (91.7) | 5,352 (87.0) | 1,636 (88.2) |
| Age, years | 43.6 ± 9.1 | 42.1 ± 10.8 | 40.7 ± 10.8 |
| eGFR, mL/min/1.73 m2 | 85.5 ± 14.9 | 91.0 ± 16.4 | 92.6 ± 16.0 |
| BMI, kg/m2 | 23.8 ± 3.4 | 23.3 ± 3.4 | 23.0 ± 3.3 |
| Smoking status, n (%) |  |  |  |
| Never | 3,492 (37.4) | 1,939 (31.5) | 517 (27.9) |
| Former | 2,081 (22.3) | 947 (15.4) | 257 (13.9) |
| Current | 3,728 (40.0) | 3,233 (52.6) | 1,072 (57.8) |
| Alcohol intake, n (%) |  |  |  |
| None | 2,435 (26.1) | 2,224 (36.2) | 654 (35.3) |
| >0 to <2 go/day | 4,754 (51.0) | 2,672 (43.4) | 814 (43.9) |
| ≥2 go/day | 2,138 (22.9) | 1,254 (20.4) | 386 (20.8) |
| Occupation |  |  |  |
| Engineering | 5,099 (54.7) | 787 (12.8) | 119 (6.4) |
| Production | 723 (7.8) | 3,451 (56.1) | 1,188 (64.1) |
| Office and administration | 2,299 (24.6) | 619 (10.1) | 79 (4.3) |
| Other | 1,005 (10.8) | 1,024 (16.7) | 409 (22.1) |
| High job position, n (%) | 2,792 (29.9) | 188 (3.1) | 19 (1.0) |
| Long overtime work (≥45 h/month) | 3,836 (41.1) | 1,701 (27.7) | 553 (29.8) |
| Shift work, n (%) | 796 (8.5) | 2,534 (41.2) | 1,076 (58.0) |
| Commuting mode, n (%) |  |  |  |
| Walking | 1,550 (16.6) | 709 (11.5) | 159 (8.6) |
| Cycling | 569 (6.1) | 387 (6.3) | 94 (5.1) |
| Bus/train (public transportation) | 2,473 (26.5) | 930 (15.1) | 225 (12.1) |
| Car/motorbike | 4,728 (50.7) | 4,120 (67.0) | 1,376 (74.2) |
| Sleep duration of <6 h/day, n (%) | 4,960 (53.2) | 2,820 (45.9) | 819 (44.2) |
| Hypertension, n (%) | 1,226 (13.1) | 855 (13.9) | 213 (11.5) |
| Diabetes, n (%) | 732 (7.8) | 501 (8.1) | 107 (5.8) |
| History of CVD, n (%) | 67 (0.7) | 34 (0.6) | 5 (0.3) |
| Dyslipidemia, n (%) | 4,670 (50.1) | 2,431 (39.5) | 643 (34.7) |
| Hyperuricemia, n (%) | 2,103 (22.5) | 1,064 (17.3) | 297 (16.0) |
| Leisure-time physical activity |  |  |  |
| Inactive (0 MET-h/week) | 5,867 (62.9) | 4,095 (66.6) | 1,208 (65.2) |
| Low (>0 to <7.5 MET-h/week) | 1,604 (17.2) | 989 (16.1) | 303 (16.3) |
| Moderate (7.5 to <16.5 MET-h/week) | 1,130 (12.1) | 594 (9.7) | 177 (9.5) |
| High (≥16.5 MET-h/week) | 726 (7.8) | 472 (7.7) | 166 (9.0) |
| Commuting physical activity, n (%) |  |  |  |
| 0 to <20 min | 4,453 (47.7) | 3,741 (60.8) | 1,188 (64.1) |
| 20 to <40 min | 3,156 (33.8) | 1,751 (28.5) | 510 (27.5) |
| ≥40 min | 1,718 (18.4) | 658 (10.7) | 156 (8.4) |
| Data are shown as the mean ± standard deviation for continuous variables and as a number (percentages) for categorical variables. The proportion of missing data for each covariate was as follows: smoking status (0.4%), occupation (3.1%), job position (3.3%), monthly overtime work (3.6%), shiftwork (2.9%), primary commuting mode (0.1%), sleep duration (0.2%), diabetes (0.2%), dyslipidemia (0.02%), and hyperuricemia (0.04%).  BMI, body mass index; CVD, cardiovascular disease; eGFR, estimated glomerular filtration rate. | | | |

**Table S2.** Baseline characteristics of participants according to commuting physical activity

|  | Commuting physical activity | | |
| --- | --- | --- | --- |
|  | 0 to <20 min | 20 to <40 min | ≥40 min |
| Participants | n = 9,382 | n = 5,417 | n = 2,532 |
| Male sex, n (%) | 8,345 (88.9) | 4,880 (90.1) | 2,319 (91.6) |
| Age, years | 41.9 ± 10.1 | 43.3 ± 9.8 | 44.8 ± 9.6 |
| eGFR, mL/min/1.73 m2 | 89.0 ± 16.0 | 87.9 ± 15.6 | 86.0 ± 15.4 |
| BMI, kg/m2 | 23.4 ± 3.4 | 23.6 ± 3.4 | 23.5 ± 3.2 |
| Smoking status, n (%) |  |  |  |
| Never | 3,035 (32.3) | 1,879 (34.7) | 1,034 (40.8) |
| Former | 1,718 (18.3) | 1,003 (18.5) | 564 (22.3) |
| Current | 4,585 (48.9) | 2,521 (46.5) | 927 (36.6) |
| Alcohol intake, n (%) |  |  |  |
| None | 3,085 (32.9) | 1,588 (29.3) | 640 (25.3) |
| >0 to <2 go/day | 4,377 (46.7) | 2,582 (47.7) | 1,281 (50.6) |
| ≥2 go/day | 1,920 (20.5) | 1,247 (23.0) | 611 (24.1) |
| Occupation |  |  |  |
| Engineering | 2,565 (27.3) | 2,187 (40.4) | 1,253 (49.5) |
| Production | 3,393 (36.2) | 1,496 (27.6) | 473 (18.7) |
| Office and administration | 1,545 (16.5) | 977 (18.0) | 475 (18.8) |
| Other | 1,617 (17.2) | 571 (10.5) | 250 (9.9) |
| High job position, n (%) | 1,232 (13.1) | 1,092 (20.2) | 675 (26.7) |
| Long overtime work (≥45 h/month) | 3,151 (33.6) | 2,016 (37.2) | 923 (36.5) |
| Shift work, n (%) | 3,064 (32.7) | 1,040 (19.2) | 302 (11.9) |
| Commuting mode, n (%) |  |  |  |
| Walking | 408 (4.3) | 1,047 (19.3) | 963 (38.0) |
| Cycling | 730 (7.8) | 247 (4.6) | 73 (2.9) |
| Bus/train (public transportation) | 566 (6.0) | 1,765 (32.6) | 1,297 (51.2) |
| Car/motorbike | 7,670 (81.8) | 2,355 (43.5) | 199 (7.9) |
| Sleep duration of< 6 h/day, n (%) | 4,599 (49.0) | 2,721 (50.2) | 1,279 (50.5) |
| Hypertension, n (%) | 1,168 (12.4) | 722 (13.3) | 404 (16.0) |
| Diabetes, n (%) | 683 (7.3) | 427 (7.9) | 230 (9.1) |
| History of CVD, n (%) | 51 (0.5) | 29 (0.5) | 26 (1.0) |
| Dyslipidemia, n (%) | 4,047 (43.1) | 2,499 (46.1) | 1,198 (47.3) |
| Hyperuricemia, n (%) | 1,791 (19.1) | 1,140 (21.0) | 533 (21.1) |
| Leisure-time physical activity |  |  |  |
| Inactive (0 MET-h/week) | 5,979 (63.7) | 3,557 (65.7) | 1,634 (64.5) |
| Low (>0 to <7.5 MET-h/week) | 1,547 (16.5) | 912 (16.8) | 437 (17.3) |
| Moderate (7.5 to <16.5 MET-h/week) | 1,070 (11.4) | 543 (10.0) | 288 (11.4) |
| High (≥16.5 MET-h/week) | 786 (8.4) | 405 (7.5) | 173 (6.8) |
| Occupational physical activity, n (%) |  |  |  |
| Sedentary | 4,453 (47.5) | 3,156 (58.3) | 1,718 (67.9) |
| Standing or walking | 3,741 (39.9) | 1,751 (32.3) | 658 (26.0) |
| Fairly active | 1,188 (12.7) | 510 (9.4) | 156 (6.2) |
| Data are shown as the mean ± standard deviation for continuous variables and as a number (percentages) for categorical variables. The proportion of missing data for each covariate was as follows: smoking status (0.4%), occupation (3.1%), job position (3.3%), monthly overtime work (3.6%), shiftwork (2.9%), primary commuting mode (0.1%), sleep duration (0.2%), diabetes (0.2%), dyslipidemia (0.02%), and hyperuricemia (0.04%).  BMI, body mass index; CVD, cardiovascular disease; eGFR, estimated glomerular filtration rate. | | | |

**Table S3**. Sensitivity analysis for the associations between leisure-time physical activities and incident chronic kidney disease.

| **Definition 1 (ours)** | | | |
| --- | --- | --- | --- |
|  | Cases/Subjects | Person-years | Adjusted hazard ratio^a^ |
| Inactive (0 MET-h/week) | 2,557/11,170 | 94,647 | 1.00 [reference] |
| Low (>0 to <7.5 MET-h/week) | 643/2,896 | 25,572 | 0.94 [0.86-1.03] |
| Moderate (7.5 to <16.5 MET-h/week) | 474/1,901 | 16,116 | 1.06 [0.96-1.17] |
| High (≥16.5 MET-h/week) | 339/1,364 | 11,417 | 1.08 [0.96-1.21] |
| **Definition 2 (Criteria recommended by the WHO^b^)** | | | |
| Inactive (0 MET-h/week) | 2,557/11,170 | 94,647 | 1.00 [reference] |
| Low (>0 to <7.5 MET-h/week) | 643/2,896 | 25,572 | 0.94 [0.86-1.03] |
| Moderate (7.5 to <14.9 MET-h/week) | 430/1,737 | 14,724 | 1.05 [0.95-1.17] |
| High (≥15.0 MET-h/week) | 383/1,528 | 12,809 | 1.08 [0.97-1.20] |
| ^a^ The model was adjusted for the same covariates as Model 3.  ^b^ The WHO recommended a minimum of 150 min/week of moderate-intensity activity or 75 min/week of vigorous or an equivalent combination (≥7.5 MET-h/week) for health benefits, and twice that level (≥15.0 MET-h/week) for additional benefits. | | | |

**Table S4.** Hazard ratios of chronic kidney disease according to occupational physical activity in subgroups.

| Subgroups | Cases/Subjects | Person-years | Occupational physical activity | | | *P* for  interaction |
| --- | --- | --- | --- | --- | --- | --- |
|  |  |  | Sedentary | Standing/Walking | Fairly active |  |
| Sex |  |  |  |  |  | 0.435 |
| Men | 3,707/15,544 | 134,509 | 1.00 [reference] | 0.90 [0.82-0.98] | 0.95 [0.83-1.09] |  |
| Women | 306/1,787 | 13,243 | 1.00 [reference] | 0.96 [0.70-1.32] | 0.84 [0.53-1.35] |  |
| Age |  |  |  |  |  | 0.375 |
| <50 years | 2,627/12,353 | 114,374 | 1.00 [reference] | 0.91 [0.81-1.01] | 0.99 [0.84-1.16] |  |
| ≥50 years | 1,386/4,978 | 33,378 | 1.00 [reference] | 0.89 [0.77-1.02] | 0.84 [0.67-1.06] |  |
| Hypertension |  |  |  |  |  |  |
| Yes | 799/2,294 | 15,989 | 1.00 [reference] | 0.91 [0.75-1.10] | 0.84 [0.62-1.14] | 0.861 |
| No | 3,214/15,037 | 131,764 | 1.00 [reference] | 0.89 [0.81-0.98] | 0.95 [0.82-1.10] |  |
| Diabetes |  |  |  |  |  |  |
| Yes | 458/1,340 | 9,338 | 1.00 [reference] | 0.89 [0.69-1.14] | 0.87 [0.60-1.40] | 0.606 |
| No | 3,543/15,956 | 138,141 | 1.00 [reference] | 0.89 [0.81-0.97] | 0.94 [0.81-1.08] |  |
| Obesity |  |  |  |  |  |  |
| BMI ≥25 kg/m^2^ | 1,541/5,054 | 41,180 | 1.00 [reference] | 0.77 [0.66-0.89] | 0.76 [0.60-0.95] | 0.150 |
| BMI <25 kg/m^2^ | 2,472/12,277 | 106,572 | 1.00 [reference] | 0.97 [0.87-1.08] | 1.03 [0.88-1.21] |  |
| Baseline eGFR |  |  |  |  |  |  |
| 60 to 89 mL/min/1.73 m^2^ | 2,843/10,248 | 84,227 | 1.00 [reference] | 0.86 [0.78-0.96] | 0.83 [0.71-0.99] | 0.090 |
| ≥90 mL/min/1.73 m^2^ | 1,170/7,083 | 63,525 | 1.00 [reference] | 0.97 [0.83-1.14] | 1.16 [0.94-1.43] |  |
| Commuting physical activity |  |  |  |  |  |  |
| <20 min | 2,013/9,382 | 80,581 | 1.00 [reference] | 0.86 [0.76-0.96] | 0.94 [0.79-1.11] | 0.319 |
| ≥20 min | 2,000/7,949 | 67,171 | 1.00 [reference] | 0.93 [0.81-1.05] | 0.91 [0.74-1.11] |  |
| Data are shown as the hazard ratio (95% confidence interval).  ^a^ Active including standing, walking, and fairly active at work.  The Cox proportional hazards regression adjusted for baseline age (continuous), sex, smoking status (never, former, or current), alcohol consumption (0, >0 to <2, or ≥2go/day), occupation (engineering, production, office and administration, or others), job position (high or low), overtime work (<45, 45 to <60, 60 to <80, 80 to <100, or ≥100 h), shift work (yes or no), primary commuting mode (walking, bicycling, train/bus, or car/motorbike), sleep duration (<5, 5 to<6, 6 to<7, or ≥7 h per day), hypertension, diabetes, history of cardiovascular disease, dyslipidemia, hyperuricemia, body mass index (<18.5, 18.5 to <25.0, 25.0 to <30.0, or ≥30.0 kg/m2), baseline estimated glomerular filtration rate (60-89 or ≥90 mL/min/1.73m^2^), leisure-time physical activity (0, >0 to <7.5, 7.5 to <16.5, or ≥16.5 MET-h/week), and walking for commuting to and from work (<20 min, 20 to <40 min, or ≥40 min). | | | | | | |

**Table S5.** Hazard ratios of chronic kidney disease according to commuting physical activity in subgroups.

| Subgroups | Cases/Subjects | Person-years | Commuting physical activity | | | *P* for  interaction |
| --- | --- | --- | --- | --- | --- | --- |
|  |  |  | <20 min | 20 to <40 min | ≥40 min |  |
| Sex |  |  |  |  |  | 0.729 |
| Men | 3,707/15,544 | 134,509 | 1.00 [reference] | 1.05 [0.96-1.14] | 1.06 [0.94-1.19] |  |
| Women | 306/1,787 | 13,243 | 1.00 [reference] | 1.18 [0.90-1.56] | 1.47 [0.98-2.21] |  |
| Age |  |  |  |  |  | 0.657 |
| <50 years | 2,627/12,353 | 114,374 | 1.00 [reference] | 1.08 [0.98-1.19] | 1.09 [0.95-1.26] |  |
| ≥50 years | 1,386/4,978 | 33,378 | 1.00 [reference] | 1.02 [0.89-1.16] | 1.07 [0.89-1.28] |  |
| Hypertension |  |  |  |  |  |  |
| Yes | 799/2,294 | 15,989 | 1.00 [reference] | 1.15 [0.96-1.37] | 1.25 [0.98-1.59] | 0.607 |
| No | 3,214/15,037 | 131,764 | 1.00 [reference] | 1.04 [0.95-1.14] | 1.04 [0.92-1.18] |  |
| Diabetes |  |  |  |  |  |  |
| Yes | 458/1,340 | 9,338 | 1.00 [reference] | 1.04 [0.81-1.35] | 0.87 [0.60-1.25] | 0.944 |
| No | 3,543/15,956 | 138,141 | 1.00 [reference] | 1.04 [0.96-1.13] | 1.08 [0.96-1.22] |  |
| Obesity |  |  |  |  |  |  |
| BMI ≥25 kg/m^2^ | 1,541/5,054 | 41,180 | 1.00 [reference] | 0.99 [0.88-1.13] | 1.03 [0.88-1.13] | 0.485 |
| BMI <25 kg/m^2^ | 2,472/12,277 | 106,572 | 1.00 [reference] | 1.10 [1.00-1.22] | 1.11 [0.97-1.29] |  |
| Baseline eGFR |  |  |  |  |  |  |
| 60 to 89 mL/min/1.73 m^2^ | 2,843/10,248 | 84,227 | 1.00 [reference] | 1.05 [0.95-1.15] | 1.04 [0.91-1.19] | 0.687 |
| ≥90 mL/min/1.73 m^2^ | 1,170/7,083 | 63,525 | 1.00 [reference] | 1.08 [0.93-1.24] | 1.17 [0.95-1.44] |  |
| Occupational physical activity |  |  |  |  |  |  |
| Sedentary | 2,403/9,327 | 79,584 | 1.00 [reference] | 1.03 [0.93-1.14] | 1.07 [0.93-1.23] | 0.490 |
| Active ^a^ | 1,610/8,004 | 68,168 | 1.00 [reference] | 1.09 [0.97-1.23] | 1.10 [0.91-1.32] |  |
| Data are shown as the hazard ratio [95% confidence interval].  ^a^ Active including standing, walking, and fairly active at work.  The Cox proportional hazards regression adjusted for baseline age (continuous), sex, smoking status (never, former, or current), alcohol consumption (0, >0 to <2, or ≥2go/day), occupation (engineering, production, office and administration, or others), job position (high or low), overtime work (<45, 45 to <60, 60 to <80, 80 to <100, or ≥100 h), shift work (yes or no), primary commuting mode (walking, bicycling, train/bus, or car/motorbike), sleep duration (<5, 5 to<6, 6 to<7, or ≥7 h per day), hypertension, diabetes, history of cardiovascular disease, dyslipidemia, hyperuricemia, body mass index (<18.5, 18.5 to <25.0, 25.0 to <30.0, or ≥30.0 kg/m2), baseline estimated glomerular filtration rate (60-89 or ≥90 mL/min/1.73m^2^), leisure-time physical activity (0, >0 to <7.5, 7.5 to <16.5, or ≥16.5 MET-h/week), and occupational physical activity (sedentary, standing or walking, and fairly physically activity). | | | | | | |

**Table S6.** Sensitivity analyses for the associations of leisure-time, occupational, and commuting physical activity with incident chronic kidney disease.

| Follow-up ≥2 years | Cases/Subjects | Person-years | Model 1 ^b^ | Model 2 ^c^ | Model 3 ^d^ |
| --- | --- | --- | --- | --- | --- |
| Leisure-time physical activity | | | | | |
| Inactive (0 MET-h/week) | 2,033/10,145 | 93,304 | 1.00 [reference] | 1.00 [reference] | 1.00 [reference] |
| Low (>0 to <7.5 MET-h/week) | 509/2,661 | 25,256 | 0.90 [0.82-0.99] | 0.91 [0.83-1.00] | 0.92 [0.84-1.02] |
| Moderate (7.5 to <16.5 MET-h/week) | 390/1,744 | 15,909 | 1.08 [0.96-1.20] | 1.08 [0.97-1.21] | 1.09 [0.98-1.22] |
| High (≥16.5 MET-h/week) | 257/1,231 | 11,244 | 0.99 [0.88-1.13] | 1.00 [0.88-1.14] | 1.02 [0.90-1.17] |
|  |  | *P* for trend ^a^ | 0.756 | 0.609 | 0.386 |
| Occupational physical activity | | | | | |
| Sedentary | 1,945/8,498 | 78,481 | **1.00 [reference]** | **1.00 [reference]** | **1.00 [reference]** |
| Standing/Walking | 967/5,593 | 51,254 | **0.85 [0.79-0.92]** | **0.85 [0.77-0.94]** | **0.87 [0.79-0.95]** |
| Fairly active | 277/1,690 | 15,978 | **0.83 [0.73-0.95]** | **0.85 [0.73-0.98]** | **0.87 [0.76-1.00]** |
|  |  | *P* for trend ^a^ | **<0.001** | **0.004** | **0.004** |
| Commuting physical activity | | | | | |
| <20 min | 1,594/8,590 | 79,543 | 1.00 [reference] | 1.00 [reference] | 1.00 [reference] |
| 20 to <40 min | 1,071/4,919 | 45,458 | 1.11 [1.03-1.20] | 1.07 [0.98-1.16] | 1.07 [0.98-1.16] |
| ≥40 min | 524/2,272 | 20,711 | 1.13 [1.03-1.25] | 1.05 [0.93-1.19] | 1.06 [0.94-1.20] |
|  |  | *P* for trend ^a^ | 0.003 | 0.297 | 0.241 |
| Data are shown as the hazard ratio [95% confidence interval].  ^a^ *P* value for the linear trend was calculated by using the Cox proportional hazards regression and assigning each category of physical activity as a continuous variable.  ^b^ Model 1 was adjusted for baseline estimated glomerular filtration rate (60 to 89 or ≥90 mL/min/1.73m^2^), age (continuous), and sex.  ^c^ Model 2 was further adjusted for baseline smoking status (never, former, or current), alcohol consumption (0, >0 to <2, or ≥2go/day), occupation (engineering, production, office and administration, or others), job position (high or low), overtime work (<45, 45 to <60, 60 to <80, 80 to <100, or ≥100 h), shift work (yes or no), commuting mode (walking, bicycling, train/bus, or car/motorbike), sleep duration (<5, 5 to<6, 6 to<7, or ≥7 h per day), and the other types of physical activity (i.e., leisure-time physical activity (0, >0 to <7.5, 7.5 to <16.5, or ≥16.5 MET-h/week), occupational physical activity (sedentary, standing or walking, and fairly physically activity), or walking for commuting to and from work (<20 min, 20 to <40 min, or ≥40 min]).  ^d^ Model 3 was further adjusted for potential mediators, including baseline hypertension, diabetes, history of cardiovascular disease, dyslipidemia, hyperuricemia, and body mass index (<18.5, 18.5 to <25.0, 25.0 to <30.0, or ≥30.0 kg/m^2^). | | | | | |

**Table S7.** Hazard ratios of chronic kidney disease according to the commuting mode.

|  | Cases/Subjects | Person-years | Model 1 ^a^ | Model 2 ^b^ | Model 3 ^c^ |
| --- | --- | --- | --- | --- | --- |
| Commuting mode | | | | | |
| Car/motorbike | 2,246 | 88,143 | 1.00 [reference] | 1.00 [reference] | 1.00 [reference] |
| Bus/train (public transportation) | 979 | 30,259 | 1.13 [1.05-1.22] | 1.07 [0.98-1.17] | 1.06 [0.97-1.17] |
| Cycling | 213 | 8,939 | 1.02 [0.89-1.18] | 1.01 [0.88-1.17] | 1.03 [0.89-1.19] |
| Walking | 574 | 20,325 | 1.11 [1.01-1.22] | 1.07 [0.98-1.17] | 1.06 [0.95-1.18] |
| Data are shown as the hazard ratio (95% confidence interval).  ^a^ Model 1 was adjusted for baseline estimated glomerular filtration rate (60 to 89 or ≥90 mL/min/1.73m^2^), age (continuous), and sex.  ^b^ Model 2 was further adjusted for baseline smoking status (never, former, or current), alcohol consumption (0, >0 to <2, or ≥2go/day), occupation (engineering, production, office and administration, or others), job position (high or low), overtime work (<45, 45 to <60, 60 to <80, 80 to <100, or ≥100 h), shift work (yes or no), commuting mode (walking, bicycling, train/bus, or car/motorbike), sleep duration (<5, 5 to<6, 6 to<7, or ≥7 h per day), and the other types of physical activity (i.e., leisure-time physical activity (0, >0 to <7.5, 7.5 to <16.5, or ≥16.5 MET-h/week), occupational physical activity (sedentary, standing or walking, and fairly physically activity), or walking for commuting to and from work (<20 min, 20 to <40 min, or ≥40 min]).  ^c^ Model 3 was further adjusted for potential mediators, including baseline hypertension, diabetes, history of cardiovascular disease, dyslipidemia, hyperuricemia, and body mass index (<18.5, 18.5 to <25.0, 25.0 to <30.0, or ≥30.0 kg/m^2^). | | | | | |
